# Predictors for Short-Term Efficacy of Allergen-Specific Sublingual Immunotherapy in Children with Allergic Rhinitis

**DOI:** 10.1155/2020/1847061

**Published:** 2020-04-21

**Authors:** Wenlong Liu, Qingxiang Zeng, Renzhong Luo

**Affiliations:** Department of Otolaryngology, Guangzhou Women and Children's Medical Center, Guangzhou Medical University, Guangzhou, China

## Abstract

**Background:**

A good compliance in allergen-specific sublingual immunotherapy (SLIT) often comes from good short-term efficacy. We aimed to evaluate the pretreatment parameters which can predict the short-term clinical efficacy in children that underwent SLIT.

**Methods:**

517 children with allergic rhinitis (AR) that underwent SLIT were recruited in this study. Baseline clinical characteristics and laboratory parameters were collected, and the clinical efficacy was evaluated using symptom and medication scores. A multivariate logistic regression model and receiver operating characteristic (ROC) curves were established.

**Results:**

A total of 303 (65%) in 466 children that underwent SLIT achieved short-term clinical efficacy. The time of using the air conditioner was negatively correlated with clinical efficacy, whereas the serum-specific IgE (s-IgE) levels, the serum IL-10 and IL-35 levels, and the s-IgE/total-IgE ratio were positively correlated with clinical efficacy.

**Conclusion:**

The time of using the air conditioner, serum-specific IgE (s-IgE) levels, serum IL-10 and IL-35 levels, and s-IgE/total-IgE ratio may be helpful for child selection before SLIT.

## 1. Introduction

Allergic rhinitis (AR) is a common pediatric chronic upper respiratory disease worldwide, which affects 10% to 20% of the children, and its prevalence has significantly increased over the last two decades [1]. Medical treatment may achieve good effect, but the improvement does not last long enough after the medicine was withdrawn.

Allergy immunotherapy (AIT) is effective for both allergic asthma and rhinitis by modifying the natural course of allergic disease and inducing allergen-specific immune tolerance [2]. The safety and efficacy of both subcutaneous immunotherapy (SCIT) and sublingual immunotherapy (SLIT) has been confirmed by several double-blind, placebo-controlled trials and meta-analyses [3–5].

Despite good efficacy, some patients discontinue or reject SLIT because the treatment is slow onset, is long-lasting, and has no surveillance like SCIT. A poor compliance is a general challenge for prolonged medical treatments. On the other side, a good compliance often comes from rapid onset and good short-term efficacy of SLIT. However, few studies have been conducted in the short-term efficacy of SLIT treatment [6].

Therefore, there is an urgent need for identifying predictors for short-term efficacy of SLIT-responsive and SLIT-nonresponsive endotypes. With these predictors, doctors can select patients who are most likely to be responsive to SLIT; thus, the SLIT efficacy and compliance could be significantly enhanced.

In this study, we report the short-term efficacy of 517 children who underwent SLIT. The clinical characteristics and laboratory parameters were analyzed for the prediction of short-term efficacy of SLIT, and receiver operating characteristic (ROC) curves were used to determine the sensitivity, specificity, and predicted values for target predictors.

## 2. Methods

### 2.1. Patients

A total of 517 children with AR that underwent SLIT between June 2017 and June 2019 were enrolled. The diagnosis of AR was defined according to ARIA criteria, and the typical symptom was recorded [1]. Allergic status was confirmed by the prick test or specific IgE (s-IgE) for Dermatophagoides farinae (Der f). We excluded the children allergic to other allergens. All the children had no history of asthma, atopic dermatitis, or other systematic diseases. The study was approved by the local ethical committee, and the written informed consent was provided.

Baseline information such as sex, age, body weight, height, parental allergic history, passive smoke history, daily diet, living environment, physical activity, disease duration, and severity were collected at the beginning of the study. The air conditioner is often used for 10 to 15 hours depending on the weather condition, and the temperature is often set between 24 and 26°C.

### 2.2. Immunotherapy

SLIT were conducted as we previously described [7]. The Der f drops were provided by Wolwopharma Biotechnology Company (Zhejiang, China). According to the manufacturer's instruction, the children were guided to take increasing doses (No. 1, 1 mg/mL; No. 2, 10 mg/mL; and No. 3, 100 mg/mL) during the first three weeks and then were instructed to have 3 drops of No. 4 solution (No. 4, 333 mg/mL) once daily during the maintenance phase. The drops were instructed to be kept under the tongue for 2-3 minutes before being swallowed. The adverse effects related to SLIT were recorded on diary cards during the whole period.

### 2.3. Symptom and Medication Score and Evaluation of Efficacy

The symptom and medication scores were assessed at baseline and 12 weeks and 6 months after SLIT. The nasal symptoms in the last two weeks of evaluation (runny nose, sneezing, blocked nose, and itchy nose) were recorded as follows: 0 = no symptoms, 1 = slight symptoms, 2 = moderate symptoms, and 3 = severe symptoms.

The children were allowed to use oral or nasal antihistamines and/or intranasal steroids in case of aggravated symptoms. The medication score was defined as the sum of medication use in the last two weeks (1 point for an antihistamine tablet, intranasal antihistamine; 2 points for an intranasal corticosteroid) according to the statement of the World Allergy Organization Task Force. The symptom and medication scores were recorded, and a combined Symptom Medication Score (SMS) was obtained by adding together the two scores.

The children that obtained a 30% SMS compared to their baseline score were defined as the response group.

### 2.4. Laboratory Parameters

Peripheral blood cell counts were measured by a Technicon-H1 blood cell counter (Bayer, Leverkusen, Germany). The serum total-IgE, eosinophil cationic protein (ECP), and s-IgE in serum were determined using a Unicap ECP kit (Pharmacia Diagnostics; Uppsala, Sweden) at baseline level and 12 weeks and 6 months after SLIT.

The serum levels of cytokines were determined by ELISA kits (R&D systems, USA) as the instructions provided by manufacturer. The sensitivity for cytokines was as follows: IL-4, 0.22 pg/mL; IL-5, 3.9 pg/mL; IL-12, 5 pg/mL; IL-13, 125 pg/mL; IL-17, 62.5 pg/mL; IL-10, 62.5 pg/mL; IL-35, 62.5 pg/mL; IFN-gamma, 8 pg/mL; and TGF-*β*, 125 pg/mL.

### 2.5. Statistical Analysis

Statistical analyses were performed with SPSS 17.0. Data were expressed as the mean and 95% confidence interval (CI) for the mean when the data distribution was normal. The two-sided *t*-test was used for group comparison. The *χ*^2^ test was used to compare categorical variables.

According to the results of the univariate analysis, the multivariate unconditional logistic regression model was used to determine the independent predicting factors for the AIT clinical response. The results were expressed as odds ratios (ORs) and their 95% CIs. For all tests, *P* < 0.05 was considered statistically significant.

## 3. Results

### 3.1. Baseline Information during SLIT

This study recruited 517 children, and the baseline information is reported in Table 1. During the six months of treatment, 51 children dropped out from the study due to different reasons (Table 2). The adverse reactions during the study are summarized in Table 3, and no severe reaction was reported.

### 3.2. Comparison between Patients with and without Short-Term Clinical Efficacy in SLIT

As shown in Table 4, the SMS after 3 and 6 months of treatment in the effective group decreased significantly. All the patients (271 children) with effective clinical responses after 3 months of treatment still reported effective clinical responses until 6 months of treatment, while 32 children received clinical responses after 6 months of treatment despite no response obtained after 3 months of treatment. In the ineffective group, the SMS was not changed after 3 and 6 months of SLIT.

Between children with and without clinical efficacy to 6 months of SLIT, significant differences were found with regard to the following characteristics: parents' educational background, time of using the air conditioner, materials for walls, serum t-IgE levels, serum s-IgE levels, serum s-IgE/t-IgE ratios, and baseline levels of IL-10, IL-35, and TGF-beta (Table 5).

### 3.3. Multivariate Analysis

Multivariate logistic regression analysis showed that the time of using the air conditioner was negatively correlated with clinical efficacy, whereas the serum-specific IgE (s-IgE) levels, the serum IL-10 and IL-35 levels, and the s-IgE/total-IgE ratio were positively correlated with clinical efficacy (Tables 6 and 7).

**Figure 1:**
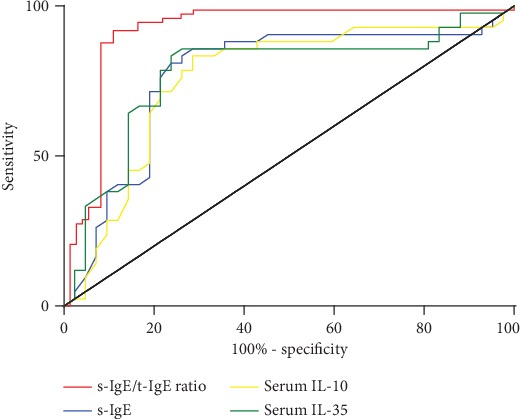
ROC curves obtained with the serum s-IgE/t-IgE ratio (decision point, 12.6%; sensitivity, 97.2%; and specificity, 74.1%), serum s-IgE levels (decision point, 34.6 IU/mL; sensitivity, 83.3%; and specificity, 74.1%), serum IL-10 levels (decision point, 98.4 pg/mL; sensitivity, 84.5%; and specificity, 72.1%), and serum IL-35 levels (decision point, 195.2 pg/mL; sensitivity, 88.9%; and specificity, 70.6%) by plotting sensitivity in children with an effective response to SLIT versus 100%-specificity in children with an ineffective response to SLIT.

Next, we determined the clinical significance of the serum s-IgE levels, s-IgE/t-IgE ratio, and levels of IL-10 and IL-35 for the prediction of short-term efficacy of SLIT. The AUC was 0.916 for the serum s-IgE/t-IgE ratio (95% CI, 0.862-0.971), 0.770 for serum s-IgE (95% CI, 0.660-0.879), 0.758 for serum IL-10 levels (95% CI, 0.648-0.868), and 0.776 for serum IL-35 levels (95% CI, 0.668-0.883) (Figure 1). When compared with each other, significant differences were found between the serum s-IgE/t-IgE ratio and the serum s-IgE (*P* < 0.01), IL-10 (*P* < 0.01), and IL-35 (*P* < 0.01). No significant difference was found between other markers. Our ROC analysis of the serum s-IgE/t-IgE ratio showed that a ratio of greater than 12.6% had the best sensitivity (97.2%) and specificity (74.1%) to predict short-term efficacy.

## 4. Discussion

The efficacy of SLIT has been proved in previous studies [4]. In general, rapid onset and good short-term efficacy often contribute to less dropout and good compliance. Therefore, it would be useful to search for specific predictors to determine those patients who might obtain rapid onset and best benefit from this therapy.

Previous studies reported inconsistent onset of action for HDM SLIT, ranging from 8 weeks to 6 months [7–9]. One randomized, placebo-controlled field trial of 120 patients that underwent SLIT assessed the onset of action as 14 weeks (the earliest measured time point was 1 week) [8]. In another field trial with 108 HDM SLIT subjects, the clinical effect on nasal symptoms appeared to begin at approximately 6 months [9]. However, the onset of action for HDM SLIT was not reported in children and most predictors were evaluated for long-term efficacy.

In the present study, we found that most children (271, 58%) received good clinical efficacy after 3 months of treatment and the response rate increased to 303 (65%) at 6 months after treatment, proving good short-term efficacy of SLIT. Besides, the treatment was safe and no severe reaction was reported.

To confirm predictors for SLIT-responsive and SLIT-nonresponsive endotypes, we compared and analyzed the clinical characteristics and laboratory parameters related to clinical efficacy. We found that parents' educational background, time of using the air conditioner, materials for walls, serum t-IgE levels, serum s-IgE levels, serum s-IgE/t-IgE ratios, and the levels of IL-10, IL-35, and TGF-beta were correlated with clinical responses. However, multivariate logistic regression analysis showed that the clinical efficacy was significantly correlated with the time of using the air conditioner, serum s-IgE levels, and levels of IL-10 and IL-35.

Previous studies showed that Der f was found and had high concentration in the air conditioner filters. Therefore, the longer the air conditioner was used, the more severe the symptoms the patients underwent, which also led to poor clinical efficacy [10].

Di Lorenzo et al.'s study reported that a significant positive correlation was found between the serum s-IgE/t-IgE ratio (>16.2) and the clinical response [11]. A pediatric study found that the serum t-IgE is superior to both the serum s-IgE/t-IgE ratio and s-IgE levels alone in predicting clinical effectiveness in children with allergic asthma and/or rhinitis due to HDM [12]. On the contrary, Fujimura et al.'s study suggested that patients with a low serum-specific IgE/total-IgE ratio (<0.19) achieved better AIT benefit than patients with a higher ratio [13]. In the present study, we found that the serum t-IgE levels, serum s-IgE levels, and serum s-IgE/t-IgE ratios were higher in the effective group compared to the ineffective group. However, multivariate logistic regression analysis showed that the clinical efficacy was only significantly positively correlated with the serum s-IgE levels and s-IgE/t-IgE ratio. We found that the serum s-IgE/t-IgE ratio is the best predictor of clinical efficacy in SLIT.

The immunotherapy has been reported to induce downregulation of the Th2 response with a shift toward a Th1 cytokine profile [14]. Moreover, upregulation of iTregs may be considered prognostic and response monitoring biomarkers, respectively, for SLIT [13]. Therefore, we analyzed the correlation of baseline cytokine levels and clinical efficacy. Our results found that baseline Th2 (IL-4, IL-5, and IL-13) and Th17 (IL-17) cytokines had no correlation with short-term efficacy, suggesting that Th1/Th2 imbalance was not reversed in the short term. Multivariate logistic regression analysis suggested baseline levels of IL-10 and IL-35 can predict clinical efficacy. Consistently, IL-35 and IL-10 can be induced by SLIT to play a protective role [15, 16]. Therefore, it is reasonable that higher baseline levels of IL-10 and IL-35 may predict good response of SLIT.

In summary, our results proved that the serum level of specific IgE (s-IgE), s-IgE/t-IgE ratio, IL-10, and IL-35 and the air conditioner use were significantly correlated with short-term efficacy of SLIT in children, which may be helpful for patient selection before SLIT.

## Figures and Tables

**Table 1:** Baseline characteristics of 466 patients that underwent SLIT.

Age (years), mean ± SEM	7.8 ± 2.5
Male/female	225/241
Duration of symptoms (years)	4.2 ± 1.1
Serum s-IgE level to Der f (IU/mL)^∗^	26.18 (10.73-68.81)
Serum s-IgE/t-IgE ratio^∗^	11.5 (10.1-15.8)
Response to SLIT	303/466

^∗^Geometric mean after logarithmic transformation (95% CI).

**Table 2:** The reasons for the dropout of 51 children during SLIT.

Reasons	Numbers
Lack of efficacy	16
Adverse events	13
Fail to take medication according to schedule	10
Loss to follow-up	12
Total	51

**Table 3:** Adverse events during SLIT.

Adverse events	Numbers
Aggravation of allergic symptom	21
Local irritation symptom	15
Gastrointestinal symptom	10
Other minor symptom	19
Total	65

**Table 4:** Clinical efficacy after short-term SLIT.

	Effective group	Ineffective group
3 months of SLIT		
Cases	271 (58%)	195 (42%)
Endpoint symptoms	2.6 ± 1.2^∗^	6.5 ± 2.7
Endpoint medication	3.7 ± 2.1^∗^	7.3 ± 3.6
SMS	7.8 ± 3.5^∗^	12.9 ± 4.3
6 months of SLIT		
Cases	303 (65%)	163 (35%)
Endpoint symptoms	2.4 ± 1.1^∗^	6.7 ± 2.8
Endpoint medication	3.1 ± 1.8^∗^	7.5 ± 3.4
SMS	6.9 ± 3.6^∗^	13.2 ± 4.6

^a^Data presented as median values (minimum-maximum). ^∗^Compared with the control group, *P* < 0.05.

**Table 5:** Univariate logistic analysis of factors associated with clinical response to SLIT.

Characteristic	Group		*P* value
	Effective	Ineffective	
Number (%)	303 (65%)	163 (35%)	0.010
Age (years)	7.3 (6.1-9.2)	7.8 (6.3-9.5)	0.134
Male (%)	170 (56.1%)	96 (58.9%)	0.204
BMI	22 (18.7-25.3)	21.5 (18.1-26.7)	0.363
Diet habits (%)			
Seafood	21.5%	20.3%	0.181
Poultry	48.1%	53.2%	0.172
Pork	75.2%	73.8%	0.346
Fast food	12.1%	11.6%	0.067
Beverages	18.5%	21.3%	0.112
Outdoor activities			
0-1 hour	34%	41%	0.231
1-2 hours	42%	41%	0.135
>3 hours	24%	19%	0.087
Bedroom time			
<12 hours	90%	85%	0.246
>12 hours	10%	15%	0.417
Living environment			
Bungalows/building	5%/95%	3%/97%	0.421
Urban/rural area	82%/18%	85%/15%	0.653
Atopic family history	57%	68%	0.512
Pet raising	8%	5%	0.214
Plush toys (%)	32%	29%	0.128
Parental smoking (%)	65%	59%	0.119
Education background (%)			
<Master's degree	38%	48%	0.013
>Master's degree	62%	52%	0.024
Air conditioning service time			
<3 months/year	21%	10%	0.006
>3 months/year	79%	90%	0.008
Bedding cleaning			
<1 time	14%	18%	0.128
>1 time	86%	82%	0.226
Wall material			
Wallpaper	11%	15%	0.019
Organic solvent coating	85%	78%	0.028
Aqueous coating	4%	7%	0.037
Floor material			
Carpet	14%	12%	0.371
Ceramic tile	63%	76%	0.446
Wood floor	23%	12%	0.257
Cooking fuel			
Coal gas	54%	42%	0.183
Oil gas	25%	30%	0.763
Natural gas	21%	28%	0.683
Duration of disease			
<1 year	33%	38%	0.051
1-2 years	21%	19%	0.117
>3 years	46%	43%	0.235
Age of onset	4 (3-6)	4.5 (3-6)	0.411
Severity			
Mild	23%	25%	0.371
Moderate	46%	48%	0.426
Severe	31%	27%	0.553
t-IgE	321.5 (178.5, 534)	413 (103, 852)	0.002
IgG4	10.4 (8.3, 11.8)	12.2 (10.9, 14.1)	0.226
s-IgE of Der f	27.7 (10.7, 68.8)	17.9 (7.3, 42.9)	0.001
s-IgE/t-IgE ratios	36.1 (21.7, 42.9)	17.8 (15.6, 21.4)	0.003
Blood leukocyte count (×10^9^/L)	5.88 (5.03-7.53)	6.02 (5.18-7.88)	0.358
Blood neutrophil count (×10^9^/L)	3.07 (2.53-3.85)	3.12 (2.68-4.05)	0.426
Blood neutrophil percent (%)	49.1 (45.3-57.6)	47.8 (48.5-59.2)	0.189
Blood lymphocyte count (×10^9^/L)	2.07 (1.69-2.78)	2.01 (1.71-2.65)	0.773
Blood lymphocyte percent (%)	33.1 (29.5-42.8)	35.9 (28.7-44.6)	0.653
Blood eosinophil count (×10^9^/L)	0.21 (0.13-0.48)	0.19 (0.11-0.38)	0.129
Blood eosinophil percent (%)	3.65 (2.34-8.67)	2.97 (1.78-6.83)	0.137
Blood monocyte count (×10^9^/L)	0.42 (0.35-0.56)	0.43 (0.33-0.59)	0.133
Blood monocyte percent (%)	7.64 (6.34-9.81)	7.42 (6.15-8.95)	0.245
Blood basophil count (×10^9^/L)	0.06 (0.03-0.11)	0.05 (0.04-0.10)	0.378
Blood basophil percent (%)	1.03 (0.85-1.04)	0.98 (0.87-1.08)	0.198
ECP (ng/mL)	40.2 (5.6-128.4)	51.3 (7.8-145.1)	0.266
IL-4 (pg/mL)	3.4 (2.1-4.5)	3.8 (1.9-4.7)	0.098
IL-5 (pg/mL)	87.3 (45.8-137.8)	73.2 (41.6-119.5)	0.178
IL-13 (pg/mL)	1256.8 (783.1-1428.3)	1198.5 (68-1428.3)	0.264
IL-12 (pg/mL)	78.2 (52.1-124.6)	67.1 (41.9-117.8)	0.073
IFN-*γ* (pg/mL)	245.1 (178.3-301.6)	218.3 (166.4-285.0)	0.133
IL-17 (pg/mL)	124.5 (86.9-147.3)	137.8 (97.8-185.2)	0.187
IL-10 (pg/mL)	92.5 (71.3-101.6)	69.3 (63.1-80.1)	0.002
IL-35 (pg/mL)	187.3 (146.5-221.8)	106.2 (85.3-127.3)	0.001
TGF-beta (pg/mL)	192.1 (132.5-223.4)	187.6 (124.6-211.7)	0.004

Mean (95% CI). ^b^Geometric mean after logarithmic transformation (95% CI).

**Table 6:** Multivariate logistic analysis of factors associated with clinical response to SLIT.

Markers	OR	95% CI	*P* value
Parents' educational background	3.478	1.251-6.095	0.13
Materials for walls	1.569	0.873-2.574	0.24
Time of using the air conditioner	2.156	1.336-3.488	0.01
t-IgE	4.825	3.119-6.085	0.35
s-IgE levels to Der f	2.673	1.983-3.249	0.01
s-IgE/t-IgE ratios	0.758	0.531-2.637	0.02
IL-10	1.239	0.642-2.745	0.03
IL-35	1.457	1.109-3.265	0.01
TGF-beta	0.875	0.668-2.188	0.07

**Table 7:** Factors associated with clinical response to SLIT.

Factors	Correlation
Time of using the air conditioner	Negative
s-IgE levels to Der f	Positive
s-IgE/t-IgE ratios	Positive
IL-10	Positive
IL-35	Positive

## Data Availability

The datasets used and/or analyzed during the current study are available from the corresponding author on reasonable request.
